# Network analysis of somatic symptoms in Chinese patients with depressive disorder

**DOI:** 10.3389/fpubh.2023.1079873

**Published:** 2023-03-13

**Authors:** Yang Li, Shoumei Jia, Baohua Cao, Li Chen, Zhongying Shi, Hao Zhang

**Affiliations:** ^1^Department of Nursing, Air Force Military Medical University, Xi'an, Shanxi, China; ^2^School of Nursing, Fudan University, Shanghai, China; ^3^Department of Nursing, Shanghai Mental Health Center, Shanghai, China; ^4^Department of Nursing, Fudan University Shanghai Cancer Center, Shanghai, China; ^5^Medical Department, The Chinese People's Liberation Army 985th Hospital, Taiyuan, Shanxi, China

**Keywords:** depression, somatic symptoms, network analysis, PHQ-15, China

## Abstract

**Introduction:**

Network theory conceptualizes somatic symptoms as a network of individual symptoms that are interconnected and influenced by each other. In this conceptualization, the network's central symptoms have the strongest effect on other symptoms. Clinical symptoms of patients with depressive disorders are largely determined by their sociocultural context. To our knowledge, no previous study has investigated the network structure of somatic symptoms among Chinese patients with depressive disorders. The aim of this study was to characterize the somatic symptoms network structure in patients with depressive disorders in Shanghai, China.

**Method:**

A total of 177 participants were recruited between October 2018 and June 2019. The Chinese version of the Patient Health Questionnaire-15 was used to assess somatic symptoms. In order to quantify the somatic symptom network structure, indicators of “closeness,” “strength,” and “betweenness” were employed as identifiers for network-central symptoms.

**Result:**

The symptoms of “feeling your heart pound or race,” “shortness of breath,” and “back pain” had the highest centrality values, indicating that these symptoms were central to the somatic symptom networks. Feeling tired or mentally ill had the strongest positive correlation with insomnia or other sleep problems (*r* = 0.419), followed by chest pain and breathlessness (*r* = 0.334), back pain, and limb or joint pain (*r* = 0.318).

**Discussion:**

Psychological and neurobiological research that offers insights into somatic symptoms may focus on these central symptoms as targets for treatment and future research.

## 1. Introduction

Approximately 3.8% of the world population is affected by depressive disorder and the total number of patients worldwide is ~280 million (1). Depression is a major contributor to death by suicide (2), and was the single most significant contributor to global disability and “non-fatal health loss,” accounting for more than 47 million disability-adjusted life years in 2019 (3).

Somatic symptoms are common among patients with depressive disorders. These are defined as physical disorders characterized by complaints of various physical discomfort symptoms that cannot be reasonably explained by the physiological disease process of experimental medicine (4). There are several distinct somatic symptoms associated with depressive disorder, including vegetative symptoms (including sleep disturbance, changes in appetite, and lack of energy), severe painful symptoms (such as headaches, backaches, stomachaches, and musculoskeletal pain), and non-painful symptoms (including palpitations, dizziness, dyspnea, and shortness of breath) (5).

According to a multicenter international study, ~66% of patients with depressive disorders were initially afflicted by somatic symptoms (6). In Western countries, the occurrence of somatic symptoms in patients with depression typically falls within the range of 66–93% (7). In China, patients with depressive disorder focus more on their somatic symptoms while ignoring shifts in emotion and frequently seek help outside of psychiatric contexts, which often leads to a higher prevalence of complaints regarding somatic symptoms, reported at 98.2% in general hospitals (7). Previous research has shown that somatic symptoms in patients with depression might indicate increased severity, worsening prognosis, worsening treatment response, chronicity, and delayed remission (8). The presence of various somatic symptoms is an important indicator of the two-year persistence of depression (OR = 1.69, 95%, confidence interval [CI] = 1.07–2.68, *p* = 0.03), as well as gastrointestinal, cardiopulmonary, and general symptomatic clusters (9). Indeed, the long-term, persistent somatic presentation of depression significantly contributes to individual experience of immense personal and familial suffering, as well as other adverse outcomes, such as functional impairment, misdiagnosis and missed diagnosis, financial burden, and increased risk of recurrence of depressive disorders (10). Somatic symptoms in patients with depression may persist even after therapy, therefore, impeding remission and raising the possibility of recurrence. Strong correlations between depression and somatic symptoms have been demonstrated with the majority of research concentrating on depression (11). Exploring the psychopathological mechanisms involved in somatic symptoms is essential to provide targeted and effective treatments.

In the common cause perspective of mental disorders, also known as the traditional theory of psychopathology, somatic symptoms are conceptualized as passive consequences of an underlying disorder (12). For example, an infection causes fever and pain in the same way that depression causes fatigue and sleep disturbances. The use of standardized depression measures in several research also suggest that somatic symptoms are seen as interchangeable manifestations of the same condition, since they tally individual answers to obtain a total score (13). The cluster of physical symptoms of depression are secondary to direct symptom-to-symptom linkages in the newly proposed psychopathology theory (i.e., the causal system view of mental diseases) (14), but they are not a common cause.Thus, somatic symptoms, such as fatigue and sleep disturbances are not the result of depression. In contrast, their effects upon one another occur through their own psychological and biological mechanisms. The existence and specificity of these interactions were identified *via* a network assessment. The key symptoms of the network showed the greatest correlation with many other symptoms. Furthermore, because central symptoms canactivate other symptoms, they may contribute heavily to the arrival and continuation of other symptoms. Therefore, it may be more efficient to target these central symptoms by focusing on biopsychosocial aspects (15). In network theory, although neighboring symptoms can activate each other, their activation can also be triggered by extrinsic circumstances, including significant and detrimental life experiences or medical health issues (14). Through network analysis, researchers can identify “bridge symptoms” that serve as catalysts for different syndromes. Therefore, compared to the classical model, the network perspective can potentially yield more clinically applicable insights into the role that early symptoms play in estimating the likelihood of future disorders.

Depressive symptoms have been analyzed using network analysis, excluding somatic symptoms, in several Western countries (16, 17). Therefore, we conducted this research with the purpose of characterizing the network structure of somatic symptoms as it applies to Chinese patients with depressive disorder.

## 2. Materials and methods

### 2.1. Setting and participants

This study was conducted in Shanghai, China, between October 2018 and June 2019. Using convenience sampling, 177 patients diagnosed with depressive disorders in the psychological outpatient department of a general hospital and the ward of a mental health hospital were invited to participate in this study. All participants fulfilled the following inclusion criteria: outpatients or inpatients aged 18 years or above whose diagnoses met the criteria for depressive disorder as defined by the International Classification of Diseases (ICD-10). The exclusion criteria were severe physical disease, schizophrenia, schizophrenic mental disorder, affective schizophrenic mental disorder, dementia, and a history of substance abuse. The study protocol was approved by the Medical Ethics Committee of the School of Nursing at Fudan University (reference number: IRB # 2018-12-06). Participants provided informed consent before participating in this research and they were assured of anonymity and confidentiality. They also retained the right to withdraw if desired.

### 2.2. Measurements

The fifteen-item Patient Health Questionnaire's (PHQ-15) Chinese version was used to quantify the somatic complaints of patients with depression. This scale may be used to screen for somatic symptoms and rate their severity. It asks questions about 15 somatic symptoms, which together comprise more than 90% of the symptoms observed in primary care (18, 19). On a three-point Likert scale, participants were asked to rate the intensity of their symptoms during the previous 4 weeks as follows: 0 (not bothered at all), 1 (bothered a little), and 2 (bothered a lot). The overall score ranged from 0 to 30, with higher values indicating more severe somatic symptoms. To clearly demonstrate the intensity of the somatic symptoms, the total score was divided into one of four categories: minimum (PHQ-15 score 0–4), mild (score 5–9), moderate (score 10–14), and severe (score 15–30) (20, 21). Cronbach's alpha of PHQ-15's Chinese version is 0.73 (22). In the current study, the Cronbach's alpha was 0.86.

Demographic information for the patients with depression included age, sex, marital status, education level, and personal monthly income in the preceding 12 months. Clinical information included stage of disease, severity of depressive disorder, and frequency.

### 2.3. Data collection

Patients with depressive disorders in the psychological outpatient department of a general hospital and the ward of a mental health hospital were investigated using questionnaires administered by two postgraduates. The investigators first explained the study's purpose, method, significance, and questionnaire to the patients. As soon as the patients provided informed consent, questionnaires were issued. The patients completed the questionnaires according to the investigators' instructions. If patients were unable to complete the form independently because of problems related to vision, education level, disease status, etc., the investigator asked the questions and filled them in a unified manner. Each questionnaire took 5–10 mins to complete, and all questionnaires were collected immediately. Following the completion of the questionnaires, the investigators examined them and asked the participants to provide any missing information. The questionnaires were regarded as invalid if all answers were identical, multiple answers were selected for the same question, or if 10% or more of the questions had not been answered.

### 2.4. Analysis

#### 2.4.1. Network assessment

In this study, we calculated the mean, standard deviation (SDs), skewness, and kurtosis for all items in the PHQ-15. A graphical Gaussian model (GGM) was used to estimate the network model (23).

Within the psychopathology network, ”node” represented every somatic symptom, and ”edge” represented the correlation between these symptoms. For network visualization, the association strength between nodes is represented by the thickness of the edges. Different colors were adopted to indicate the correlation direction (i.e., the red edge indicates a negative correlation and the blue edge indicates a positive correlation).

The Extended Bayesian information criteria were combined with the graphical Least Absolute Shrinkage and Selection Operator model (gLASSO) (24) in order to reduce the number of spurious edges and improve the stability of the network. Given that this study's sample size was limited, the parameters were set to γ 0.1 to delete edges with small or unstable correlations between entries, and to ensure that an accurate network could be estimated (25). The Fruchterman-Reingold algorithm was employed to draw the network diagram, and the nodes with more or stronger connections were placed at the center of the diagram, while the nodes with fewer connections were placed at the outer edge of the diagram. Network estimation was performed and visualized using the R software “qgraph” package (26).

#### 2.4.2. Identification of central symptoms

In order to determine which symptoms in the somatic symptom network were most important, three key centrality indices (strength, betweenness, and closeness) were assessed (26). Strength, the most important centrality metric in this study, was defined as the absolute sum of the edge weights associated with a node, indicating the relevance of a certain element. The more this node might influence the entire network, the higher its strong centrality score. As opposed to closeness, which was calculated as the reciprocal of the sum of a node's distance from every other node in the network, betweenness was computed as the frequency of a node lying on all the shortest pathways between other nodes (26). The analyses were performed using the “IsingFit,” “networktools,” and “qgraph” packages in R (version 3.6.3).

#### 2.4.3. Estimation of the stability and accuracy of the network

Three approaches were employed to assess the accuracy and stability of the network model and gauge the robustness of the findings. First, the 95% confidence interval (CI) was calculated using the non-parametric bootstrap technique to determine the correctness of edge weights. The projected edge weight was more accurately predicted by a network with a tighter CI than that with a broader CI (26). Second, the subset bootstrap approach was used to assess the stability of the centrality indices using the correlation stability coefficient (CS-C) (27). If the node centrality indices do not substantially change after eliminating any samples from the dataset, the network topology may be assumed to be stable. The CS-C should be at least 0.25 and better than 0.5. The differences between the two strength indices were deemed statistically significant only when the 1000-bootstrap 95% non-parametric CIs did not include zero. Bootstrapped difference tests were used to assess variations in network characteristics (28). To evaluate whether there was a significant difference between the two edge weights or two node centrality indices, the test used 95% CIs.

### 2.5. Relationship between the mean levels of symptoms, the variability, and the centrality index

The centrality indices and average scores of the PHQ-15 items, as well as the centrality indices and symptom SDs, were determined using Spearman's rank-order correlation. While the correlation between centrality indices and SDs was used to determine whether the centrality of symptoms could be attributed to the differential variability of the items, the correlation between centrality indices and the average score of PHQ-15 items was used to determine whether the most central symptoms were the most serious (16).

## 3. Results

### 3.1. Sociodemographic characteristics of participants

A total of 181 responses were collected, of which 177 were valid. The sociodemographic attributes and other clinical characteristics of the participants are presented in Table 1.

**Table 1:** Sociodemographic and clinical characteristics of participants (*N* = 177).

**Variables**	***N***	**%**
**Gender**		
Male	61	34.5%
Female	116	65.5%
**Marital status**		
Married/cohabiting	91	51.4%
Unmarried or divorced	83	46.9%
Widowed	3	1.7%
**Education level**		
Elementary or below^a^	31	17.6%
High school or secondary specialized school	72	41.8%
College or higher	74	40.7%
**Personal monthly income**		
Less than 2,000 RMB (approx. <$289)	58	32.8%
2,000–5,000 RMB (approx. $289–723)	53	29.9%
More than 5,000 RMB (approx. more than $723)	66	37.2%
Age (years)	38.41	16.15
**Stages of disease**		
Acute phase	120	67.8%
Consolidation phase	35	19.8%
Maintenance phase	22	12.4%
**Severity of depressive disorder**		
Mild	110	62.1%
Moderate	41	23.2%
Severe	26	14.7%
**Frequency**		
1 time	81	45.8%
≥2 times	96	54.2%
PHQ-15 total score	7.73	4.959

^a^Elementary or below = <7 years of education.

### 3.2. Somatic symptoms of patients with depressive disorder

Detailed information on the mean, SD, skewness, and kurtosis of somatic symptoms based on the PHQ-15 is presented in Table 2. According to the PHQ-15, the mean (SD) total score was 7.73 ± 4.959. The symptom “fainting spells” received the lowest mean score, whereas the item “feeling fatigued or having little energy” had the highest mean score.

**Table 2:** Item description of somatic symptoms as measured by the PHQ-15 (*N* = 177).

**Somatic symptoms**	**M**	**SD**	**Min**	**Max**	**Skewness**	**Kurtosis**	**% (Absence)**	**% (Presence)**
1. Back pain	0.42	0.696	0	2	1.351	0.390	69.5	30.5
2. Pain in arms, legs, or joints (knees, hips, etc.)	0.24	0.544	0	2	2.238	3.973	81.9	18.1
3. Menstrual cramps or other problems with periods (women only)	0.34	0.603	0	2	1.563	1.341	72.3	27.7
4. Headaches	0.50	0.724	0	2	1.080	−0.265	63.3	36.7
5. Fainting spells	0.08	0.335	0	2	4.269	18.793	93.2	6.8
6. Pain or problems during sexual intercourse	0.21	0.521	0	2	2.410	4.853	83.6	16.4
7. Chest pain	0.24	0.503	0	2	1.985	3.173	79.1	20.9
8. Shortness of breath	0.51	0.683	0	2	0.975	−0.279	59.3	40.7
9. Feeling heart pounding or racing	0.67	0.765	0	2	0.635	−1.018	50.8	49.2
10. Dizziness	0.70	0.750	0	2	0.555	−1.030	47.5	52.5
11. Feeling tired or having low energy	1.45	0.760	0	2	−0.954	−0.613	16.4	83.6
12. Trouble sleeping	1.29	0.827	0	2	−0.582	−1.293	23.7	76.3
13. Stomach pain	0.18	0.441	0	2	2.440	5.470	84.2	15.8
14. Nausea, gas, or indigestion	0.37	0.619	0	2	1.442	0.946	70.1	29.9
15. Constipation, loose bowels, or diarrhea	0.50	0.716	0	2	1.070	0.251	62.7	37.3

M, mean; Min, minimum; Max, maximum; PHQ-15, Patient Health Questionnaire-15; SD, standard deviation.

### 3.3. Analysis of the structure of the network and the centrality measure

**Figure 1:**
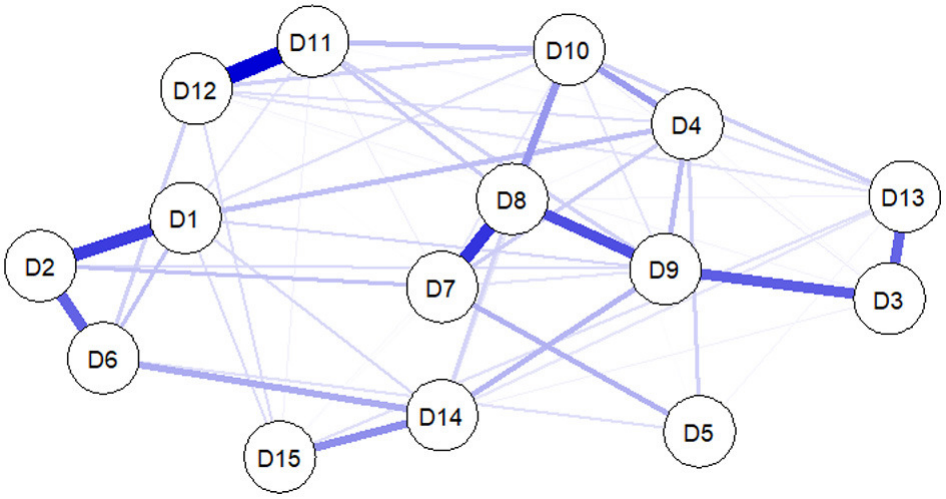
Estimated network plot for symptoms in the total sample. The network model was estimated using the GGM model. D1, back pain; D2, pain in arms, legs, or joints; D3, menstrual cramps or other problems with periods; D4, headaches; D5, fainting spells; D6, pain or problems during sexual intercourse; D7, chest pain; D8, shortness of breath; D9, feeling heart pounding or racing; D10, dizziness; D11, feeling tired or having low energy; D12, trouble sleeping; D13, stomach pain; D14, nausea, gas, or indigestion; D15, constipation, loose bowels, or diarrhea.

Figure 1 shows the network of somatic symptoms calculated using the GGM. Chest discomfort, shortness of breath, and feeling the heart pound or race were all grouped as nodes in the middle of the figure, indicating that they were all intimately related to the other symptoms in the network. Additionally, there were significant positive associations between feeling exhausted or low in energy and having problems sleeping, as well as between chest discomfort and shortness of breath, back pain, and joint, arm, or leg pain. The numerical relationships between symptoms were investigated using a weighted adjacency matrix.

**Figure 2:**
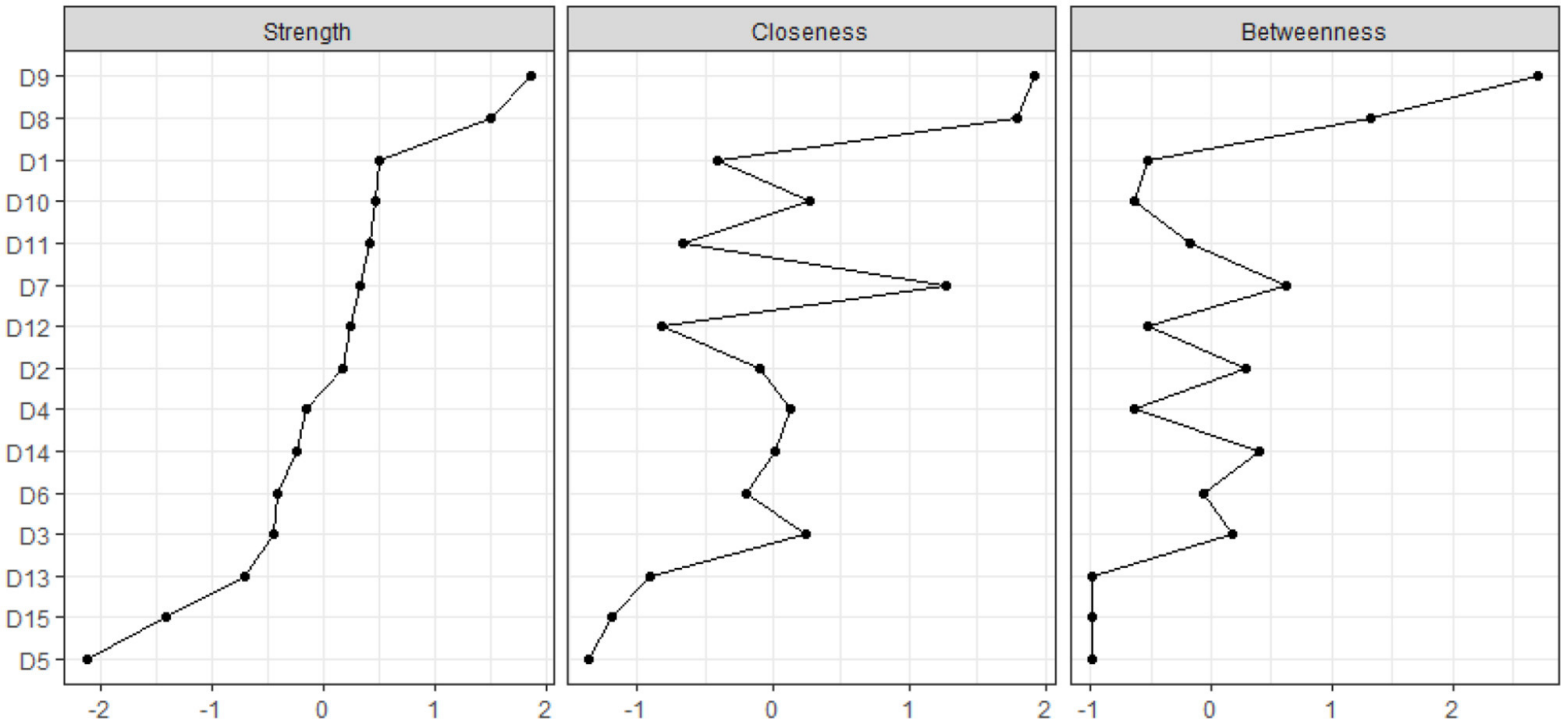
Centrality measures of all symptoms within the network. The figure shows the centrality measures (i.e., strength, betweenness, and closeness) of all factors within the network (z-scores).

Figure 2 displays the network's strength, betweenness, and closeness centrality metrics for each symptom. The symptoms of racing or pounding of the heart were the strongest, closest, and most intense, followed by back discomfort and shortness of breath. Shortness of breath and back discomfort followed the indications of a quick heartbeat or acceleration, which were the strongest across a wide area.

### 3.4. Stability and accuracy of the network

**Figure 3:**
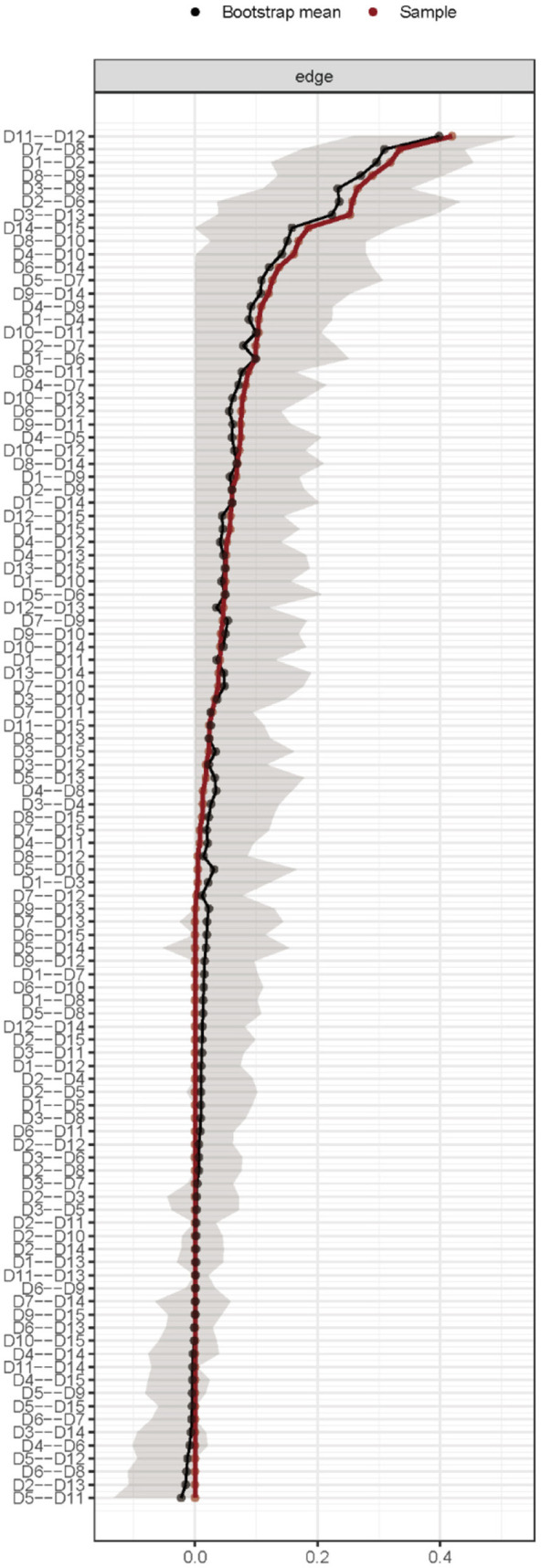
Edge accuracy plot depicting 95% confidence obtained from 2,500 bootstrap samples.

The gray area in Figure 3 represents the 95% CI area of the edge weight obtained using the bootstrap method. The 95% CIs (gray intervals) for the edge weights were small, indicating that the edge weights were accurate through network analysis and that the edges estimated by the entire network were stable.

**Figure 4:**
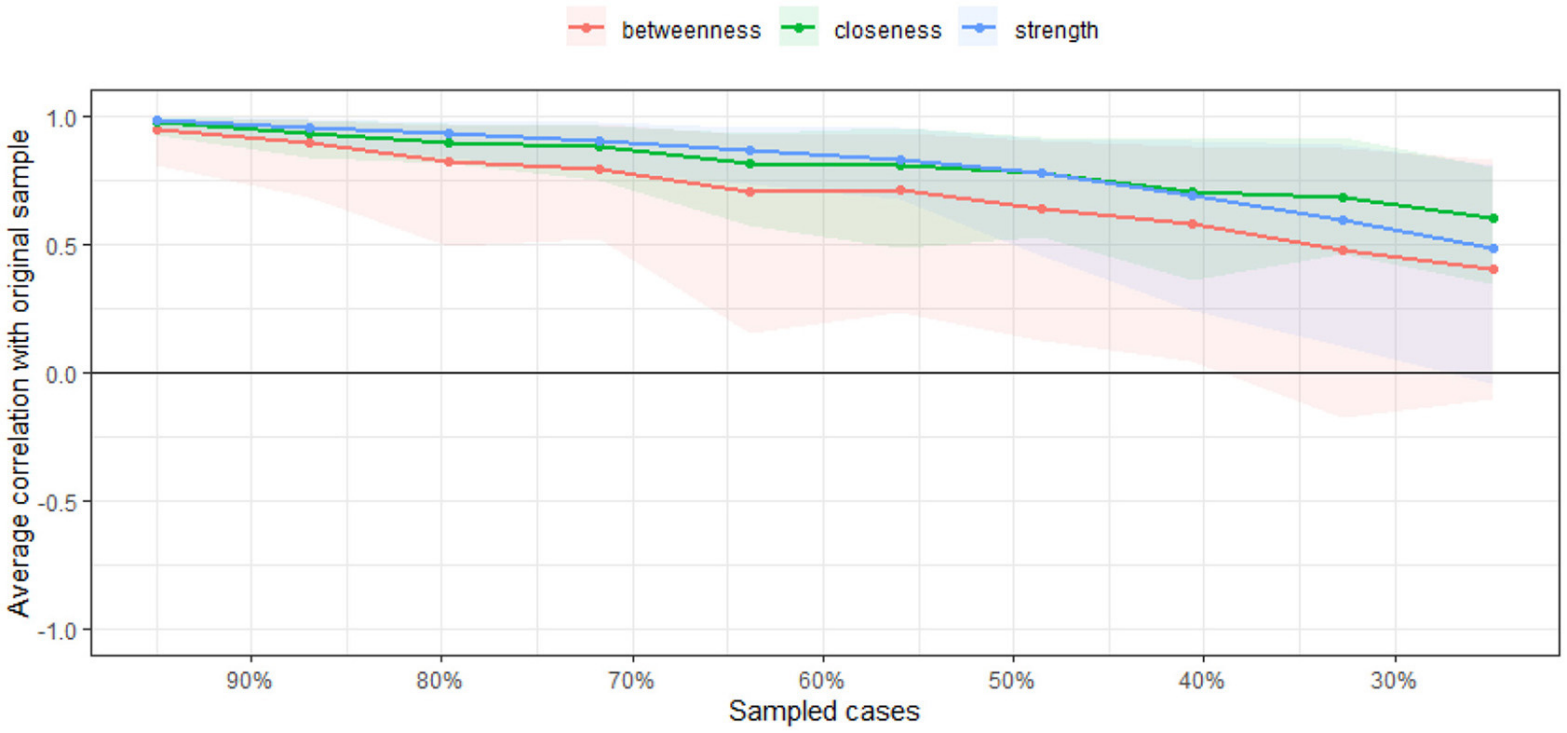
Correlation stability plot measuring the stability of strength.

The case-dropping subset bootstrap approach demonstrated that the values of strength, betweenness, and closeness were consistent even after significant chunks of the sample were dropped. Although betweenness had a little lower stability than the primary one (CS-C = 0.051), while closeness had a better stability. In contrast, this sample's strength index was solid and trustworthy (CS-C = 0.441), which was relatively stable and could be used to explain the importance of symptoms (Figure 4). Therefore, we primarily focused on interpreting symptom strength using network analysis.

**Figure 5:**
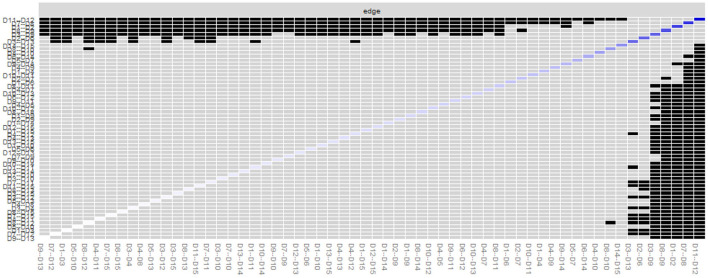
Bootstrapped difference test for edge weights.

In Figure 5 black squares indicate edges that do differ significantly from one-another, whereas gray squares indicate edges that do not differ significantly from one-another. It is found that the edges with strong connections, such as D11–D12, D07–D08, D01–D02, are significantly different from most other edges in the network structure, while the remaining edges with lower edge weights have no significant difference from one-another.

**Figure 6:**
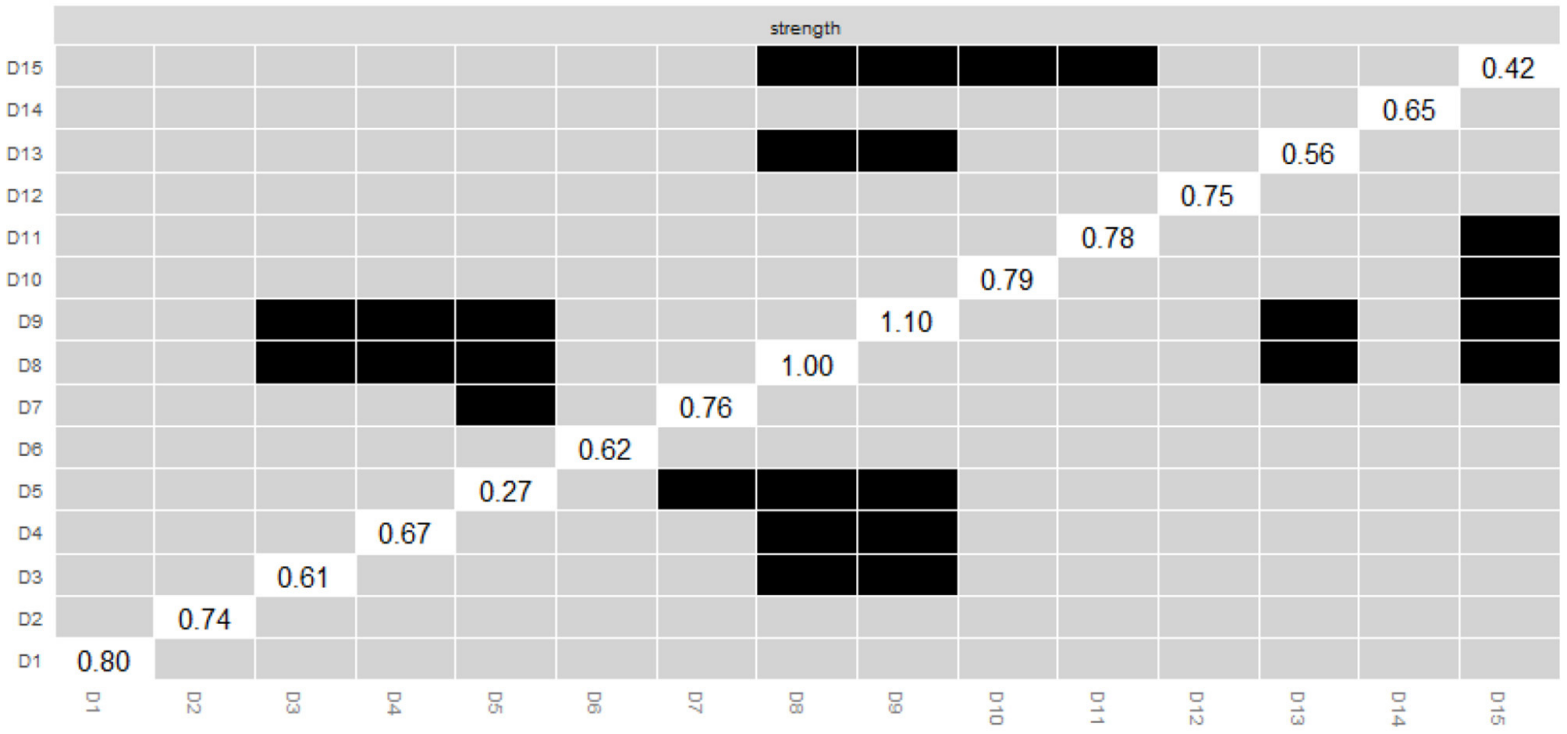
Estimation of node strength difference by bootstrapped difference test. Bootstrapped difference tests between-node strength of factors. Gray boxes indicate nodes that do not significantly differ from one another. Black boxes represent nodes that differ significantly from one another (α = 0.05). White boxes show the values of node strength.

Figure 6 shows that there was no difference in intensity centrality among some symptoms in the network estimation. However, D9 (feeling heart pound or race) and D8 (shortness of breath), the two most intense centrality symptoms, are different from other symptoms.

## 4. Discussions

This study determined that feeling one's heart pound or race was ranked as the most prominent somatic symptom, accompanied by shortness of breath and back pain. These three symptoms were arranged in the center of the diagram, suggesting they are highly connected with the remaining somatic symptoms and could maintain or trigger them in this sample. This result is consistent with the research of Yang Xiangyun on 1,497 outpatients in general hospitals, which showed that cardiothoracic discomfort scores (PHQ-15 score ranging from 10 to 14) in the group with multiple somatic symptoms were significantly higher than those without multiple somatic symptoms, indicating that the presence of cardiothoracic discomfort felt by patients would affect the occurrence of somatic symptoms in patients with depression (29). This may be related to the high mental stress caused by somatic symptoms and hyperthermia of the hypothalamus-pituitary-adrenal axis (HPA) caused by depressive symptoms (30). Hyperfunction of the HPA axis leads to feeling one's heart pound or race (heart palpitations) and shortness of breath (chest tightness), which are some of the most easily perceived and worrying physical manifestations among Chinese patients (31). Patients regard these two symptoms as manifestations of heart disease, so they use more medical services (such as repeating visits to a cardiology department or conducting various cardiac examinations) and avoid daily activities, which will cause patients' health anxiety – that is, causing them to pay too much attention to physical symptoms and promoting the emergence of other symptoms (32). In clinical practice, more attention should be focused on palpitations and chest tightness in patients with depressive disorder, and efforts increased toward early identification and targeted model management to ensure improvement in the somatic symptoms and treatment outcomes (7, 32).

Back pain is also considered one of the most common causes of patients seeking medical care in primary care and emergency settings, costing $200 billion annually in the United States (33). Influenced by Chinese culture, patients with depressive disorders tend to pay more attention to pain than emotional problems (32). Suicidal ideation in patients with low back pain was significantly greater than that in patients without comorbid conditions (31, 34). In a single patient, pain and depression can be attributed to one or more factors in various ways. First, the physical and psychological discomfort caused by chronic pain interacts with social and personal vulnerability to accelerate the onset of depressive episodes (35). Second, depression can be a precursor to and sometimes a contributor to pain. Major depression reduces an individual's ability to tolerate pain, and physical discomfort can be a prominent symptom. It should be noted that more than half of patients with depression consider pain as their main symptom when seeking primary care (36).

Feeling tired or mentally ill had the strongest positive correlation with insomnia or other sleep problems (*r* = 0.419), followed by chest pain and breathlessness (*r* = 0.334), and back pain, and limb or joint pain (*r* = 0.318). According to one study, there is a clear correlation between fatigue and sleep disorders, the more serious the sleep disorder, the more obvious the sense of fatigue (37). Long et al. (38) explored the causal model of fatigue symptoms in lung cancer patients and found that the greatest impacting factors on the degree of fatigue in patients were dyspnea, cough, and insomnia, of which insomnia was the most important factor that directly led to fatigue. In addition, sleep disorders can also mediate fatigue symptoms experienced by patients; Brown et al. reported that 35% of fatigue symptoms were caused by sleep disorders (39). Chest pain and breathlessness belong to the cardiothoracic discomfort symptom group, whereas back pain and limb joint pain belong to the pain symptom group, suggesting that various factors in the same symptom group affect each other and jointly promote the occurrence and development of symptoms. Attention should be paid to the synergy between symptoms during follow-up interventions.

### 4.1. Limitations

The present study has some limitations. First, the cross-sectional information used to construct the network structure of depression-related somatic symptoms among patients cannot provide a detailed picture of symptom development over time nor can it determine the causal relationship between symptoms. In future studies, longitudinal follow-up should be conducted to explore the temporal causal relationship between symptoms. Second, the network structure in this study is specific to patients with depressive disorders in Shanghai, China. As different social and cultural backgrounds impact the symptom network, the study's results cannot be extended to the national population of patients with depressive disorders. Finally, this study evaluated the somatic symptom network structure at the patient group level, which may be different from the somatic symptom network structure at the individual level.

### 4.2. Conclusion

A network analysis was conducted to construct somatic symptoms of patients with depressive disorders, providing a supplementary method for the classification and dimension model of traditional mental disorders. This research is one of the few studies to explore the relationship between somatic symptoms in patients with depressive disorders using network methods, providing new insights for better understanding the functional relationship between somatic symptoms and the clinical significance of specific symptoms. Specifically, this study found that heart palpitations, chest tightness, and back pain constitute the “backbone” that sustained the somatic symptom structure among Chinese patients with depressive disorders, therefore, suggesting that considering these symptoms as the core target may help to further reduce the overall somatic symptom severity. Fatigue had the strongest positive correlation with insomnia or other sleep problems, followed by chest pain and breathlessness, back pain, and limb or joint pain, suggesting that attention should be paid to the synergy between symptoms during follow-up interventions.

## Data availability statement

The raw data supporting the conclusions of this article will be made available by the authors, without undue reservation.

## Ethics statement

This study was approved by the Medical Ethics Committee of the Fudan University School of Nursing (reference number: IRB # 2018-12-06). The participants provided informed consent prior to their participation in this study.

## Author contributions

Study design and manuscript drafting: YL and SJ. Data collection: YL and LC. Analysis and interpretation: YL and HZ. Critical revision of the manuscript: SJ, ZS, and BC. All authors approval of the final version for publication.
